# Utilization of radiotherapy and hypofractionated radiotherapy in Japan: long-term trends and the influence of COVID-19 pandemic

**DOI:** 10.1093/jrr/rraf088

**Published:** 2026-01-30

**Authors:** Kazuya Takeda, Rei Umezawa, Takaya Yamamoto, Noriyoshi Takahashi, Shinsaku Okuda, Katsumasa Nakamura, Keiichi Jingu

**Affiliations:** Department of Radiation Oncology, Tohoku University Graduate School of Medicine, 1-1 Seiryo-machi, Aoba-ku, Sendai, Miyagi 980-8574, Japan; Department of Radiation Oncology, South Miyagi Medical Center, 38-1 Nishi, Ogawara, Shibata-gun, Miyagi, 989-1253, Japan; Department of Radiation Oncology, Tohoku University Graduate School of Medicine, 1-1 Seiryo-machi, Aoba-ku, Sendai, Miyagi 980-8574, Japan; Department of Radiation Oncology, Tohoku University Graduate School of Medicine, 1-1 Seiryo-machi, Aoba-ku, Sendai, Miyagi 980-8574, Japan; Department of Radiation Oncology, Tohoku University Graduate School of Medicine, 1-1 Seiryo-machi, Aoba-ku, Sendai, Miyagi 980-8574, Japan; Department of Radiation Oncology, Tohoku University Graduate School of Medicine, 1-1 Seiryo-machi, Aoba-ku, Sendai, Miyagi 980-8574, Japan; Department of Radiation Oncology, Hamamatsu University School of Medicine, 1-20-1 Handayama, Chuo-ku, Hamamatsu, Shizuoka, 431-3192, Japan; Department of Radiation Oncology, Tohoku University Graduate School of Medicine, 1-1 Seiryo-machi, Aoba-ku, Sendai, Miyagi 980-8574, Japan

**Keywords:** radiotherapy, COVID-19, Japan, hypofractionated radiotherapy, breast cancer, prostate cancer

## Abstract

During the coronavirus disease 2019 (COVID-19) pandemic, a short-term decline in radiotherapy use was reported. In this study, we investigated long-term changes in the use of radiotherapy and hypofractionated radiotherapy before and after the COVID-19 pandemic period in Japan and assessed whether the pandemic influenced the adoption of hypofractionation. We obtained data for analysis from the National Database Open Data for fiscal years (FYs) 2014 to 2023. For the 5 years from FY2019 to FY2023, with detailed monthly data available, we used interrupted time series (ITS) analysis to examine changes in the level and slope of claims at the boundaries of the baseline, pandemic and post-pandemic periods. During the 10-year study period, the number of radiotherapy claims increased by an average of 1.6% per year. In FY2020, the peak of the COVID-19 pandemic, the number of radiotherapy claims decreased by 2.0% compared to the previous year. The number of claims for extra fees for hypofractionated radiotherapy for breast and prostate cancer showed a steady increase, except for a temporary decline around the April 2022 policy change in payment requirements for prostate cancer. In the ITS analysis, there were no significant changes in the level and slope of claims for radiotherapy and extra fees for hypofractionated radiotherapy at the onset of the pandemic. In conclusion, the impact of the COVID-19 pandemic on the utilization of radiotherapy in Japan was minimal, and the use of hypofractionated radiotherapy showed a steady increase throughout this period.

## INTRODUCTION

The rapid spread of coronavirus disease 2019 (COVID-19) since the first reported case in late 2019 led to a global pandemic in early 2020. During the pandemic, cancer diagnosis and access to cancer care were reduced due to redeployment of staff and inpatient capacity to infectious-disease care, temporary ward closures during outbreaks and restrictions on outpatient visits [1–4]. It was reported that the annual number of radiotherapy fractions in Japan declined by 5.8% compared to the number in the previous year [5]. Additionally, there was an increase in the use of hypofractionated radiotherapy during the pandemic period, with the aim of reducing hospital visits and facilitating treatment completion [4]. It was reported that insurance claims for hypofractionated radiotherapy for breast cancer and prostate cancer during the pandemic period increased by 28.6 and 74.8%, respectively, compared with the previous year [5]. However, there is a lack of long-term studies on the utilization of overall and hypofractionated radiotherapy in Japan, and it is unclear whether these changes were due only to the COVID-19 pandemic or to other long-term trends.

The purpose of this study was to describe long-term changes in the utilization of overall and hypofractionated radiotherapy for breast and prostate cancer in Japan and to assess whether the COVID-19 pandemic influenced the radiotherapy case numbers and adoption of hypofractionated radiotherapy, using an insurance claims database.

## MATERIALS AND METHODS

### Data source and case definition

In this study, we investigated the total claim numbers of radiotherapy courses and hypofractionated radiotherapy extra fee in postoperative radiotherapy for breast cancer and definitive radiotherapy for prostate cancer. For this purpose, we obtained data from the National Database (NDB) Open Data. NDB Open Data is a publicly available database containing information on insurance claims in Japan. We accessed the homepage of the Japanese Ministry of Health, Labour and Welfare (https://www.mhlw.go.jp/stf/seisakunitsuite/bunya/0000177182.html; last accessed 1 June 2025) and downloaded the dataset.

From this database, we extracted data for the external beam radiotherapy and the corresponding surgical procedures, as listed in Table 1. To calculate the number of claims for overall radiotherapy and for hypofractionated radiotherapy, we tabulated the claims for the following items: (i) radiotherapy management fees for radiotherapy and radiotherapy fees for special radiotherapy such as stereotactic radiation therapy, which can be claimed only once or twice per one treatment course, (ii) extra fees for hypofractionated radiotherapy in postoperative radiotherapy for conservative breast cancer surgery, which can be claimed for each treatment session and (iii) extra fees for hypofractionated radiotherapy in definitive radiotherapy for prostate cancer, which can be claimed for each treatment session. For comparison, we also collected claims data for (iv) breast cancer surgery and (v) prostate cancer surgery, totaling five categories for analysis.

**Table 1:** Adopted medical action codes from NDB Open Data

Treatment content	Code	Medical action code
Radiotherapy	A400	190197910, 190198010
	M000	113001110, 180018410, 180018510, 180019010, 180019110, 180019210, 180019310, 180031710
	M001-2	180018910
	M001-3	180026750, 180019710, 180035310
	M001-4	180046610, 180046710, 180055010, 180055110, 180055210, 180055310
	M002	180012710
Extra fee for hypofractionated radiotherapy	(Breast)	180043270
	(Prostate)	180054970
Breast surgery for malignant neoplasm	K476	150121610, 150121710, 150121810, 150121910, 150122150, 150316510, 150386410, 150386510, 150262710, 150303110
	K476-5	150444250
Prostatectomy for malignant neoplasm	K843	150209310
	K843-2	150326510
	K843-3	150338810
	K843-4	150390310

### Study period and time classification

The fiscal year (FY) in Japan starts in April, and we extracted data from FY2014 to FY2023. Monthly data were also available from FY2019, and we obtained these monthly data from April 2019 to March 2024. There were some differences between the annual case numbers and the total numbers from the monthly data because small numbers of <10 were masked in the datasets for protecting personal information.

To assess the impact of the COVID-19 pandemic, we analyzed the monthly data for the five medical procedures by dividing them into three periods. The first state of emergency in Japan was declared on 7 April 2020, and the last nationwide measure, the priority measures to prevent the spread of disease, ended on 21 March 2022. In this study, we defined this interval as the COVID-19 pandemic period in Japan. Based on this, the 12 months from April 2019 to March 2020 were defined as the baseline period, the 24 months from April 2020 to March 2022 as the pandemic period and the 24 months from April 2022 to March 2024 as the post-pandemic period. At the April 2022 boundary, in addition to the end of the priority measures to prevent the spread of disease, the requirements for the extra fee for hypofractionated prostate cancer radiotherapy became more stringent (the requirement of the minimum dose per fraction was raised from 2.5 to 3.0 Gy), and we also considered this effect.

### Statistical analysis

To evaluate long-term trends in the use of radiotherapy, we tabulated annual claims data for all radiotherapy procedures, hypofractionated radiotherapy and surgeries for prostate and breast cancer.

We used interrupted time series (ITS) analysis [6] on monthly series from April 2019 to March 2024 to evaluate the impact of the COVID-19 pandemic outbreak in March–April 2020 and the end of various restrictions in March–April 2022 on the number of claims for the five medical procedures. For each procedure, we calculated the relative change over time, with the number of claims in April 2019 as the baseline of 100. To evaluate the immediate change in the number of claims (level change) and the change in the trend of medical practice over time (slope change) at the time of the pandemic outbreak and the relaxation of restrictions, we adopted a segmented regression model with change points for both the intercept and the slope. Considering the insufficiency of the length of the baseline period and the characteristics of the diseases and treatments, a correction model for seasonal variation was not used. The model was implemented and analyzed using R 4.2.2 (The R Foundation for Statistical Computing, Vienna, Austria). As this was a descriptive study, no adjustment for multiple testing was made, and a *P*-value of <0.05 was considered statistically significant.

### Use of AI tools

The artificial inteligent (AI) tools ChatGPT (https://chatgpt.com/), Google AI Studio (https://aistudio.google.com/) and Anthropic Claude (https://www.anthropic.com/claude) were used to optimize the analysis codes and assist in preparing the initial draft of the manuscript for this study. After using these tools, the authors carefully reviewed and corrected the content manually.

## RESULTS

### Annual trends in claims

The annual changes in the number of radiotherapy claims are shown in Table 2. During the 10-year study period, the total number of radiotherapy claims gradually increased by an average of 1.6% per year. In FY2020, the pandemic period in Japan, the number of radiotherapy claims decreased by 2.0% compared to FY2019. For surgery, the number of claims for malignant neoplasm surgeries of the breast and prostate decreased by 6.1 and 4.6%, respectively, compared to the previous year. While the number of claims for breast and prostate cancer surgery recovered rapidly in FY2021, with increases of 6.7 and 6.0%, respectively, the increase in radiotherapy claims in the same year was 0.9%. Consequently, while the number of claims for breast and prostate cancer surgery returned to the pre-pandemic FY2019 levels in FY2021, the recovery of radiotherapy claims was delayed until FY2022.

**Table 2:** Number of insurance claims

	Radiotherapy		Extra fee for hypofractionated radiotherapy				Surgery for malignant tumor			
	Total cases		Breast cancer		Prostate cancer		Breast cancer		Prostate cancer	
FY2014	261 954		–		–		82 121		20 254	
FY2015	267 115	(+2.0%)	66 737		–		89 559	(+9.1%)	22 977	(+13.4%)
FY2016	272 034	(+1.8%)	83 699	(+25.4%)	–		91 235	(+1.9%)	21 867	(−4.8%)
FY2017	271 952	(−0.0%)	95 091	(+13.6%)	–		90 520	(−0.8%)	22 857	(+4.5%)
FY2018	280 773	(+3.2%)	122 299	(+28.6%)	29 802		91 902	(+1.5%)	22 728	(−0.6%)
FY2019	291 235	(+3.7%)	159 925	(+30.8%)	50 753	(+70.3%)	98 179	(+6.8%)	24 172	(+6.4%)
FY2020	285 490	(−2.0%)	204 455	(+27.8%)	87 923	(+73.2%)	92 205	(−6.1%)	23 061	(−4.6%)
FY2021	288 046	(+0.9%)	241 107	(+17.9%)	111 211	(+26.5%)	98 392	(+6.7%)	24 449	(+6.0%)
FY2022	293 701	(+2.0%)	305 249	(+26.6%)	79 848	(−28.2%)	100 936	(+2.6%)	27 804	(+13.7%)
FY2023	301 157	(+2.5%)	370 201	(+21.3%)	111 217	(+39.3%)	105 712	(+4.7%)	28 469	(+2.4%)

For hypofractionated radiotherapy, extra fees were approved in FY2015 for breast cancer and in FY2018 for prostate cancer, after which the number of claims increased. The number of claims for the extra fee for hypofractionated radiotherapy for breast cancer showed an increasing trend throughout the 9-year period studied, with an average annual increase rate of 24.0%, including during the COVID-19 pandemic. The number of claims for the extra fee for hypofractionated prostate radiotherapy showed a steeper increase but temporarily decreased following the change in payment requirements in April 2022 (a 68% decrease in April 2022 compared to March 2022). However, the number of claims for the extra fee for hypofractionated prostate radiotherapy subsequently began to increase again, and the number of claims in FY2023 recovered to nearly the same level as in FY2021.

### ITS analysis of the impact of COVID-19 and policy change

**Fig. 1:**
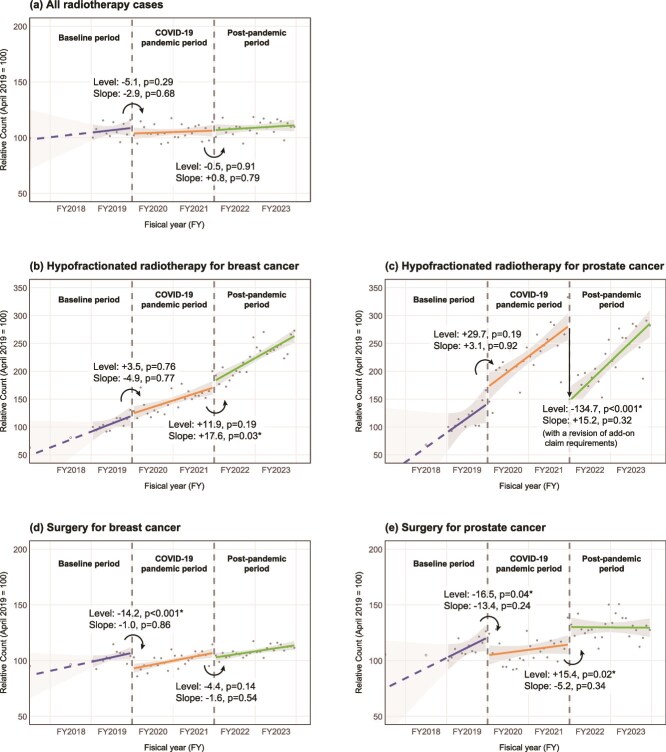
Interrupted time series (ITS) analysis for medical claim numbers. The shaded areas represent the 95% confidence interval of the regression line. Asterisks (^*^) indicate statistical significance at *P* < 0.05. For FY2017 and FY2018, annual data are shown as dashed dots and were not included in the ITS analysis.

Figure 1 shows the results of the ITS analysis, which examined changes in the number of claims (relative values with the number of claims in April 2019 set to 100) and their trends before and after the transition between the three analysis periods: baseline, pandemic and post-pandemic. At the onset of the pandemic in Japan in April 2020, the trends in claims for breast and prostate cancer surgery showed a significant drop in level (breast cancer: −14.2 points, *P* < 0.001; prostate cancer: −16.5 points, *P* < 0.001), suggesting a pandemic-induced decrease. However, the changes in the levels were not significant for the number of radiotherapy claims (−5.1 points, *P* = 0.29) or the number of claims for the extra fee for hypofractionated radiotherapy for breast and prostate cancer (breast cancer: +3.5 points, *P* = 0.76; prostate cancer: +29.7 points, *P* = 0.19). Regarding the changes following the end of the priority measures to prevent the spread of disease in late March 2022, a significant increase in the level of claims was observed for prostate cancer surgery (+15.4 points, *P* = 0.02), suggesting an increase in surgical cases with the easing of restrictions. No discontinuity in the level was observed at this point for breast cancer surgery, overall radiotherapy or the extra fee for hypofractionated breast radiotherapy. However, for the extra fee for hypofractionated breast radiotherapy, an increase in the slope was observed after this point (+17.6 points/year, *P* = 0.03). For the extra fee for hypofractionated prostate radiotherapy, which faced both the end of the priority measures to prevent the spread of disease and a more stringent payment requirement in April 2022, a large drop in the level was observed at this point (−134.7 points, *P* < 0.001). There was no change in the slope before and after this point (+15.2 points/year, *P* = 0.32), and an increasing trend was observed again after April 2022.

## DISCUSSION

The COVID-19 pandemic posed a huge challenge to cancer care globally in early 2020 as medical resources were concentrated and usual outpatient care was restricted. Staff and inpatient capacity including intensive care units were redeployed to infectious-disease care, and some wards were temporarily closed due to outbreaks, which also affected inpatient capacity. These changes reduced opportunities for cancer diagnosis and treatment, including surgery and radiotherapy [1–4]. In terms of radiotherapy, each country, professional society and facility took action to minimize the negative impact on cancer care [4, 7–10]. The actions included infection control measures in clinics, staffing optimization and the use of hypofractionated radiotherapy in curative and palliative radiotherapy. However, many of the existing reports focused on the short-term impacts on treatment cases for several months after the start of the pandemic. To address this issue, we conducted a study using long-term insurance claims data from NDB Open Data from 2014 to 2023, including the periods of the COVID-19 pandemic in Japan. Japan is a universal health insurance state in which most of the population is covered by health insurance. Therefore, the database coverage of NDB was extremely high, and we could analyze comprehensive nationwide data over a long period in this study. This is the first study to investigate the utilization of overall and hypofractionated radiotherapy in Japan over a long period of time including before and after the COVID-19 pandemic, thereby providing both frontline clinicians and policymakers with essential information for future pandemic preparedness.

Regarding the use of radiotherapy during the pandemic, there was a 2.0% decrease in the number of claims in FY2020 compared to the previous year. However, in the ITS analysis, which considered time-series trends, the decrease in the level of radiotherapy claims due to the COVID-19 pandemic outbreak was not significant, in contrast to the significant level decreases observed for breast and prostate cancer surgery claims. This could be partly due to the independent nature of radiotherapy departments, in contrast to surgery which faced limited capacity of inpatient wards and intensive care units for postoperative care in the pandemic period. The 2.0% decline in radiotherapy usage in Japan was relatively mild compared to reports from other countries, which showed an 8–10% decrease in radiotherapy cases during the first 12 months of the pandemic [11, 12]. It is known that Japan has a decentralized distribution of relatively small-scale radiotherapy facilities compared to other countries [13], and this healthcare system may have contributed to minimizing the loss of treatment opportunities during the pandemic when free movement of the patients was restricted.

Although the reduction in radiotherapy claims was relatively small, their recovery to pre-pandemic FY2019 levels did not occur until FY2022. Considering that the number of breast and prostate cancer surgery cases rapidly recovered to pre-pandemic FY2019 levels in FY2021, it is conceivable that the utilization of radiotherapy in Japan experienced a sustained, albeit small, impact. In Japan, some breast cancer patients avoided postoperative radiotherapy due to unsubstantiated media reports claiming that it caused immunosuppression and severe COVID-19 infection, although the Japanese Society for Radiation Oncology (JASTRO) issued statements denying this. Thus, public concern may have delayed the recovery in the use of radiotherapy.

The increased use of hypofractionated radiotherapy in Japan in FY2020, associated with the COVID-19 pandemic, was previously reported. Tamari *et al*. reported that the number of stereotactic radiotherapy courses increased by 15.1% in FY2020, while the number of conventional radiotherapy courses decreased by 4.0% [5]. They also noted that outpatient claims for hypofractionated radiotherapy for breast and prostate cancer increased by 28.6 and 74.8%, respectively, compared to the previous year. In May 2020, JASTRO issued official recommendations for radiotherapy during a pandemic, which included recommendations on the use of hypofractionation and infection control [14]. These efforts may have contributed to the increased use of hypofractionated radiotherapy during the pandemic. However, the ITS analysis of our study showed that while no significant change in the level of claims for hypofractionated radiotherapy was observed around the start of the COVID-19 outbreak, a consistent upward trend persisted. Furthermore, for hypofractionated breast radiotherapy, the slope of claims even steepened in the post-pandemic period. This may be because hypofractionated radiotherapy, with its guaranteed clinical equivalence, has been continuously favored post-pandemic due to a preference for patient convenience and medical efficiency [15, 16]. Furthermore, the reduction in treatment duration resulting from hypofractionation could have served as a financial incentive for high-volume centers, enabling greater patient throughput and an expansion of treatment capacity. The spread of hypofractionation has indeed led to a reduction in the burden of hospital visits for patients. According to public data from the Japanese Radiation Oncology Database, a nationwide registry led by JASTRO, the average number of treatment days for registered patients decreased from 32.6 days for breast cancer and 38.9 days for urologic cancer in FY2015 to 28.4 and 30.9 days, respectively, in FY2022 [17].

Changes in treatment strategies during the pandemic varied widely across countries. Reports from the UK showed that the use of hypofractionated radiotherapy, 26 Gy in five fractions, increased rapidly from <1% in February 2020 to over 60% in April 2020, following the release of National Health Service (NHS) guidance [18, 19]. In the UK, policy declarations from public authorities appeared to be effective in immediately changing the treatment strategy nationwide. In contrast, the changes in the use of hypofractionated radiotherapy in Japan were relatively gradual, similar to reports from the USA and the Netherlands [20, 21].

For hypofractionated prostate radiotherapy, it was difficult to estimate the effects of the end of COVID-19 restrictions and the change in payment requirements separately, as these two events occurred simultaneously at the discontinuity point of the ITS analysis in March–April 2022. However, considering the analysis results for overall radiotherapy and the extra fee for hypofractionated breast radiotherapy, it is unlikely that the end of COVID-19 restrictions caused a major drop in the claims for the extra fee for hypofractionated prostate radiotherapy. Therefore, the majority of the impact seemed to be due to the change in payment requirements. Despite the fact that the number of claims per patient decreased due to fewer fractions with a higher dose per fraction, the number of claims for the extra fee for hypofractionated prostate radiotherapy recovered by the end of FY2023 to a level comparable to that of FY2022. This suggests that individual facilities promptly adapted to the increase in dose per fraction, based on evidence of the efficacy and safety of moderate hypofractionation [22, 23].

This study has several limitations due to the available dataset. First, due to the nature of the dataset, we were unable to determine the precise total number of radiotherapy courses for postoperative breast cancer and prostate cancer. This means that a potential decrease in the overall number of breast and prostate cancer cases in FY2020 because of the COVID-19 pandemic could have made the relative proportion of hypofractionated radiotherapy appear to increase more sharply. Second, the number of data points in the baseline period for the ITS analysis was relatively small (12 observation points), as monthly data were only available from FY2019. This may have reduced the stability of the data and limited the examination of seasonal variations, but the baseline regression model showed no obvious contradiction when extrapolated to the annual data up to FY2018, suggesting that the overall validity of the analysis was maintained. Third, this study could not account for complex variations such as treatment delays or changes in treatment modality for individual patients, or interactions between treatment modalities during the pandemic. This requires a more detailed investigation of individual patient treatment behavior using individual data from the NDB in future studies.

In conclusion, we revealed long-term changes in the use of overall and hypofractionated radiotherapy. The impact of the COVID-19 pandemic on radiotherapy in Japan was small, and hypofractionated radiotherapy for postoperative breast cancer and definitive prostate cancer showed a steady increase throughout the study period.

## PRESENTATION AT A CONFERENCE

This work was presented in part at the 84th Annual Meeting of the Japan Radiological Society, Yokohama, 2025.
